# Gravity-Induced Symmetry Breaking in Chemical Gardens

**DOI:** 10.1021/acsomega.4c10551

**Published:** 2025-01-28

**Authors:** Martina Costa Reis

**Affiliations:** School of Engineering, University of São Paulo, 05508-010 São Paulo, Brazil

## Abstract

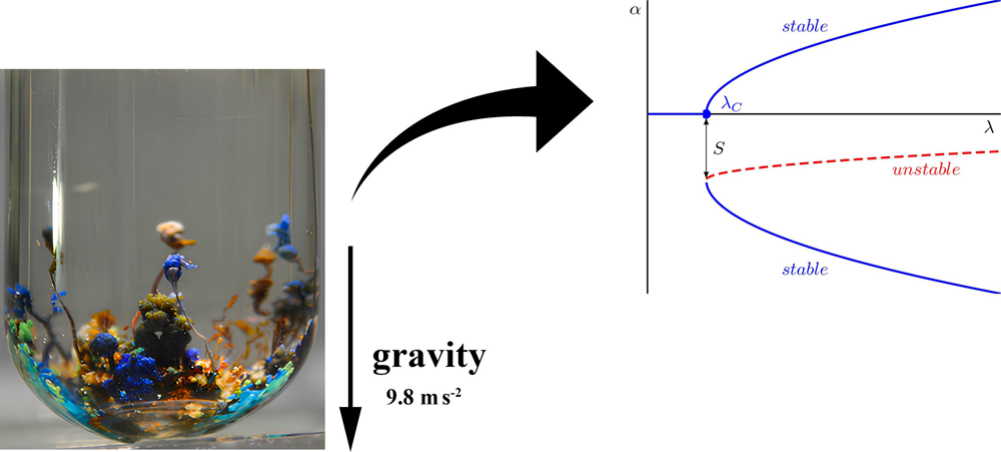

Chemical gardens are hollow precipitates with a plant-like appearance formed when a metal salt seed is immersed in an alkaline aqueous solution containing silicate, phosphate, or carbonate ions. Due to their potential to mimic biological and geological structures relevant to the understanding of life’s emergence on Earth and Mars, the study of the nonequilibrium properties of chemical gardens has become increasingly important. Hence, in this article, the influence of gravity on the formation and growth of chemical gardens is investigated. To this end, experimental evidence of the influence of gravity on the formation and growth of chemical gardens is analyzed according to nonequilibrium sensitivity theory. The results obtained from the nonequilibrium sensitivity analysis show that the upward-growing pattern observed in chemical gardens, usually formed under Earth’s gravity, is a consequence of symmetry breaking in the system’s bifurcating solutions. Under these circumstances, the thermal fluctuations within the system become negligible, favoring the vertical growth of the chemical garden. Moreover, by exploiting the definition of nonequilibrium sensitivity, the minimum magnitude of the gravitational field necessary for the vertical growth of a chemical garden was estimated. The results indicate that the upward growth pattern emerges as the dominant dissipative structure for gravitational field magnitudes larger than 10^–5^ m s^–2^, provided fluctuations remain negligible.

## 1 Introduction

**Figure 1:**
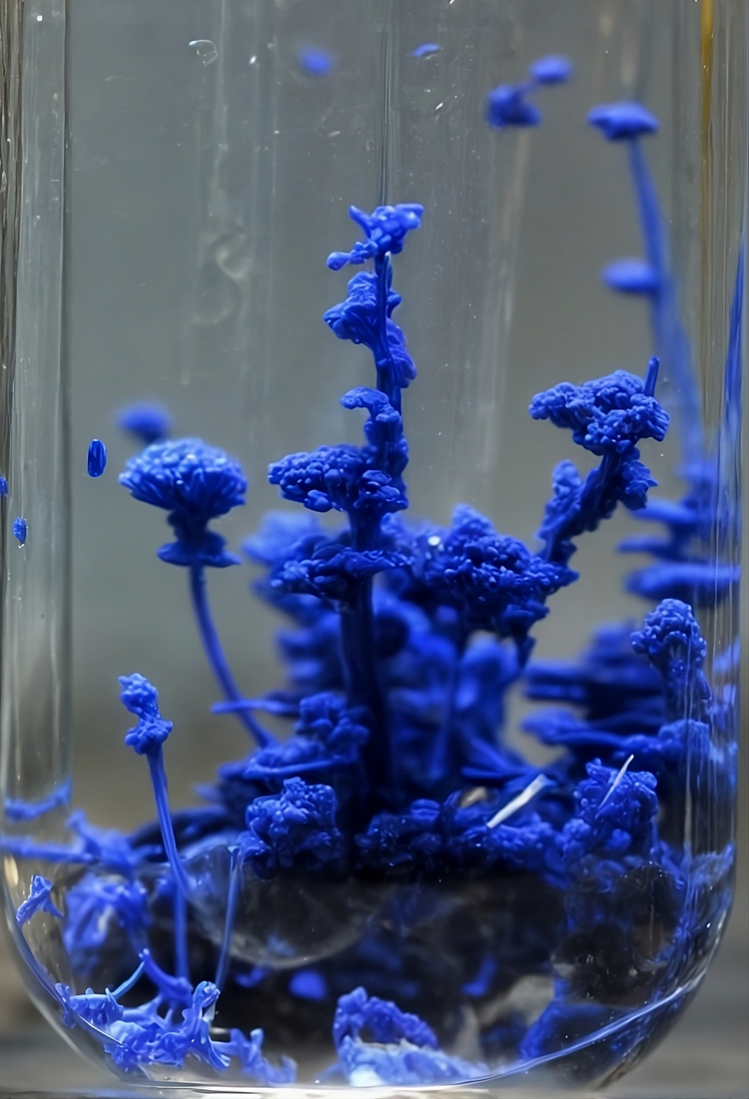
Silicate chemical gardens grown under normal gravity conditions. The morphological aspect observed is typical of chemical gardens obtained by the seed growth method.

Although the first reports of chemical gardens date back to the 17th century, they were not studied in detail until the 2000s.^1^ Chemical gardens, also known as silica gardens or crystal gardens, are permanent, self-organizing structures formed when a metal salt seed is dropped into an alkaline aqueous solution containing anions such as silicate, phosphate, or carbonate.^2^ If the solution is left undisturbed, within a few minutes, a tree-like precipitate is produced, as illustrated in Figure 1.

While interest in chemical gardens was previously restricted mainly to science fair projects, nowadays they have become a hot research topic with direct applications in the development of new materials and technologies.^3−5^ These applications include the preparation of cementitious materials, understanding the structure of corroded steel surfaces, and the development of biomedical devices for drug delivery, among others. Given this panorama, various synthesis methods for chemical gardens have naturally emerged, leading to the organization of a chemobrionics database,^6^ which categorizes chemical gardens according to the nature of the ions involved and the experimental protocol used.

Among the methods used to synthesize chemical gardens in aqueous medium, the seed growth and flow-injection methods are the two most cited approaches in the literature.^7^ The flow-injection method stands out due to its capability to control the growth of chemical gardens under well-known experimental conditions. According to this method,^8,9^ a syringe pump is employed to feed a solution of a metal salt into a reservoir containing sodium silicate solution at a previously determined flow rate. By doing so, one can reproducibly obtain chemical gardens that resemble upward-growing tubes and investigate the effect of pH, nature and concentration of ions, and even gravity on the morphology and composition of the precipitates. However, the seed growth method is the most common method to form chemical gardens. Unlike the flow-injection method, in the seed growth method, chemical gardens are formed after immersion of small crystals of a metallic salt in a solution of sodium silicate at rest.^10^ Because no experimental control is imposed on the system, the chemical gardens formed via the seed growth method tend to exhibit a very irregular morphological aspect.

Actually, the interest in the formation and growth of chemical gardens under microgravity (μ**g**) and normal gravity (1**g**) conditions stems from the fact that chemical gardens are regarded as analogues of hydrothermal vents—structures from which geothermally heated water is expelled.^11−15^ In mid-2017, NASA’s Mars Reconnaissance Orbiter collected evidence of hydrothermal vents on the Martian surface, whose origin dates back 3.7 billion years ago, precisely when the earliest life forms found on Earth emerged. Therefore, scientists speculate that, if Earth and Mars shared the same conditions 3.7 billion years ago, life might also have been possible on Mars.^16^

Notwithstanding the increasing interest in the formation and growth of chemical gardens under micro- and normal gravity conditions, very little is known about the subject. In fact, there are only a few publications^17−19^ in the literature that report the formation and growth of metal silicate precipitates under microgravity conditions. According to these papers, when compared with the precipitates formed under normal gravity, the precipitates obtained in a microgravity environment grow randomly, and their formation is much slower, suggesting that gravity plays an important role in the selection of dissipative structures.

Hence, in this work, the influence of the gravitational field on the formation and growth of chemical gardens formed via the seed growth method is examined through the nonequilibrium sensitivity theory.^20^ To this end, the chemical garden is regarded as a chemical system sufficiently far from equilibrium whose spatiotemporal evolution is described by a solution-diffusion model with reaction. The results obtained from the nonequilibrium sensitivity analysis show that the gravitational field plays a role that goes beyond forming a concentration gradient in the medium. Under microgravity conditions, thermal fluctuations are predominant near the critical point, making the system insensitive to the gravitational field. However, under 1**g** conditions, the strength of the gravitational field is sufficient to favor the upward growth pattern. To quantify these conclusions, the degree of asymmetry between the bifurcating states that emerge as the system passes through a critical point is numerically evaluated. This quantity, also called the nonequilibrium sensitivity of the chemical garden to gravitational fields, indicates that the upward growth pattern will be the most probable dissipative structure as long as the gravitational field magnitude is larger than .

## 2 Experimental Evidence for the Influence of Gravity on the Formation and Growth of Chemical Gardens

**Figure 2:**
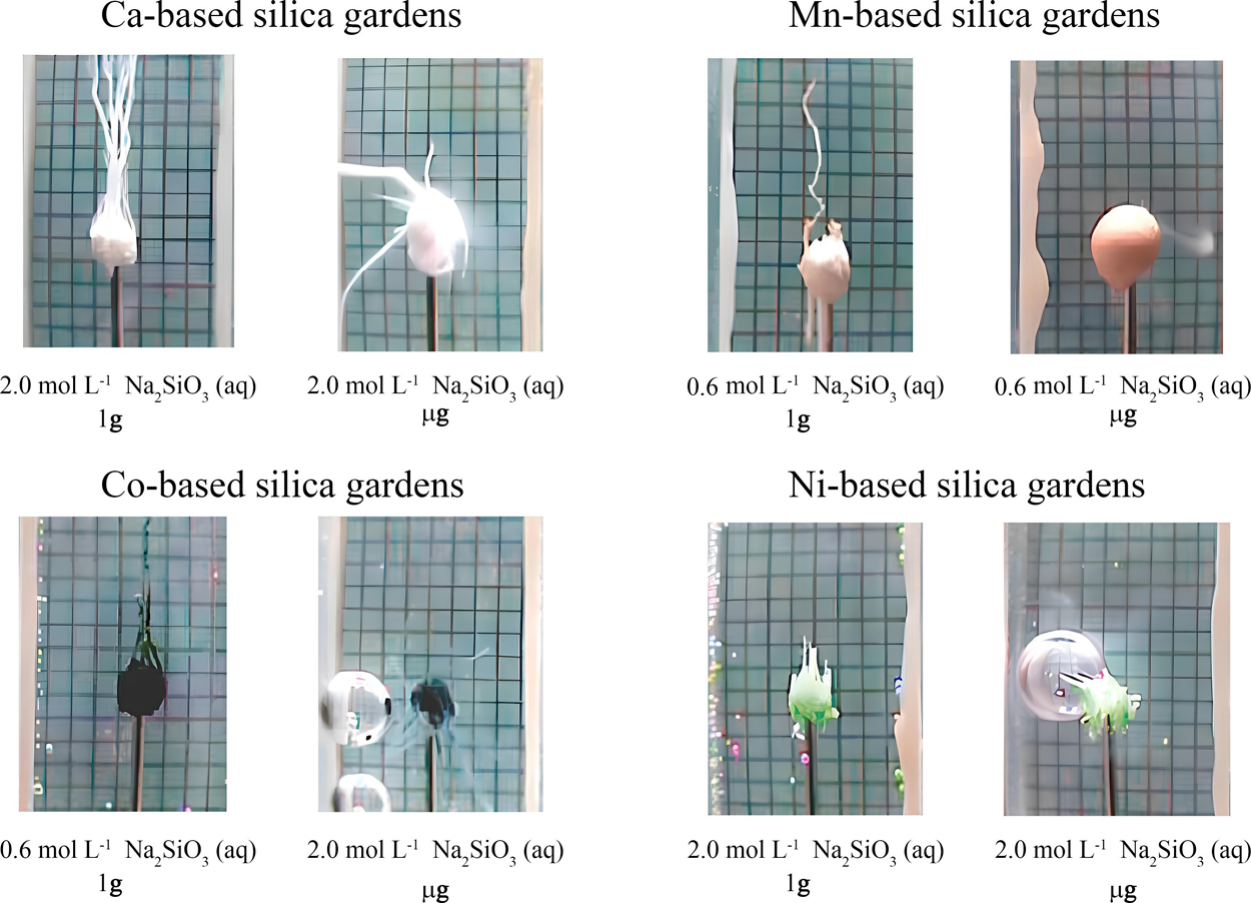
Comparison between silicate chemical gardens grown under ground and microgravity conditions via the seed method. The microgravity experiments were conducted aboard the International Space Station (ISS) during the space shuttle Endeavor mission. In all experiments, a mixture of metal salt and epoxy glue was set in a rod-shaped tube placed inside a chamber filled with sodium silicate solutions at concentrations ranging from 0.6 to 2.0 mol L^–1^. When the apparatus was activated, a device was used to push the metal salt into the chamber. For ground experiments, images of the chemical gardens were captured after 8 h of growth, while under microgravity conditions, images were taken in situ 5 days after the experiments began.^18^ Adapted with permission from Langmuir 2011, 27, 7, 3294–3300. © 2011 American Chemical Society.

Usually, chemical gardens grown under 1**g** conditions are quite different from those grown under microgravity. While chemical gardens under normal gravity conditions tend to grow upward, those formed in microgravity environments grow randomly without any preferred direction, as illustrated in Figure 2. Moreover, Cartwright et al.^18^ and Sainz-Díaz et al.^19^ also noted that chemical gardens formed in microgravity environments tend to grow more slowly than those formed on Earth. For example, while the formation of chemical gardens under normal gravity conditions may take only a few minutes, under microgravity, the formation of chemical gardens may last for several days.

The experimental evidence reported above suggests that the gravitational field plays a significant role in the formation and growth of chemical gardens. In fact, as soon as the seed of a metallic salt is immersed in a solution of sodium silicate, one observes the formation of a thin semipermeable membrane of metal silicate around the seed of the metallic salt. Then, water flows from the sodium silicate solution into the membrane, leading to an increase in osmotic pressure in its interior and eventually causing the membrane to rupture. When the membrane bursts, solution rich in metallic ions is expelled toward the sodium silicate solution and, since the metal ion solution is less dense than the sodium silicate solution, a reaction front propagates upward due to the buoyancy-driven flow of ions.^1^ As a result, one has the formation of predominantly vertical tubes of metal silicates. Moreover, the buoyant forces due to the differences in the density of sodium silicate and metallic salt solutions result in convective currents that can enhance mass transport over long distances in a short time scale, which explains why chemical gardens grow faster under 1**g** conditions.

On the other hand, under microgravity conditions, mass transport is predominantly governed by osmosis and diffusion. Here, one also has the formation of the thin semipermeable membrane of metal silicate around the seed of the metallic salt and the osmotic flow of water into the interior of the membrane. However, because the mass transport is mainly due to the diffusive forces, the reaction front does not exhibit any preferred propagation direction and the chemical garden tends to grow randomly. Moreover, the mass transport due to diffusive forces is much slower than that observed for convective forces. In fact, because of the inherent random movement of ions in a diffusive regime, the time required for significant mass transport increases and, consequently, the growth rate of the chemical gardens is limited by the diffusive flux of ions.

## 3 Overview of the Nonequilibrium Sensitivity Theory

According to the experimental accounts given in the previous section, chemical gardens are indeed thermodynamically sensitive to the gravity field. Although the concept of nonequilibrium sensitivity might appear new, it dates back to the 1980s, when Kondepudi, Prigogine, and Nelson developed, in a series of papers,^20−23^ a theoretical framework that allows one to understand how nonequilibrium thermodynamic systems may be affected by external fields and how such external fields can break the bifurcation symmetry of the system.

Thus, for the sake of simplicity, assume that a chemical garden consists of two boundary layers separated by a sufficiently dense metal silicate membrane, so that the pressure within the membrane is uniform and equal to the pressure in the sodium silicate solution. The water flux is proportional to the difference between the transmembrane hydraulic pressure (Δ*p*) and the transmembrane osmotic pressure (ΔΠ), whereas the solute flux is proportional to the gradient of concentration. In addition, consider that the concentration of ions is also influenced by the gravitational field and precipitation chemical reactions, with no-flux boundary conditions imposed at both the bottom and top boundaries of the system to allow for the breaking of the system’s reflection symmetry.^24^ Under these conditions, one has
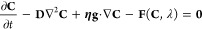
1where **C** = (*c*_1_, *c*_2_,...,*c*_*n*_) is a column vector representing the concentration of the ions, **D** and **η** are, respectively, the matrices of the diffusion and mobility coefficients, **g** is the gravitational field, **F**(**C**, λ) is a nonlinear term that expresses the mass production due to a chemical reaction, and λ is the bifurcation parameter that can be taken as a physicochemical constraint that holds the system far from the equilibrium.

To investigate the influence of the gravitational field on the formation and growth of chemical gardens, it suffices to analyze the behavior of the steady-state solutions of eq 1 near a bifurcation point, where a small gravity-induced bias can lead the system to a state of broken symmetry. In this case, one has
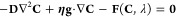
2

According to eq 2, three different dissipative mechanisms—diffusion, buoyant mass transport, and chemical reactions—may push the system far from equilibrium. While diffusive flux tends to homogenize the concentration of the species and is isotropic, the mass production term due to chemical reactions tends to enhance the fluctuations in concentration and is invariant under spatial inversion and time translation. In turn, the buoyant mass transport is not invariant under spatial inversion but can enhance the fluctuations in concentration. Mathematically, such aspects may be stated in terms of group symmetry concepts, or more precisely, the covariance of the dissipative mechanisms with respect to a symmetry group operator.

Hence, let eq 2 be rewritten as
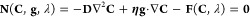
3where **N**(**C**, **g**, λ) is a nonlinear operator. Moreover, assume that the symmetry group  is defined by the identity **I** and spatial inversion **T** operators, such that  and **TT**^–1^ = **I**. If one applies the spatial inversion operator onto **N**(**C**, **g**, λ), one has **TN**(**C**, **g**, λ) = **N**(**TC**, **Tg**, λ) = **N**(**C**, – **g**, λ), which means that the gravitational field breaks the symmetry of the solutions of **N**(**C**, **g**, λ).

To better understand the symmetry breaking of the solutions of eq 3 due to the gravitational field, consider the steady-state solution of the nonlinear operator **N**(**C**, **g**, λ) at the critical value λ_C_,
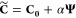
4where **C**_**0**_ is the homogeneous solution obtained for **F**(**C**_**0**_, λ) = **0**, α is the time-dependent bifurcation amplitude, and **Ψ** is the corresponding eigenmode. One can show that the bifurcation amplitude α must satisfy the following reduced bifurcation equation^25^ whose derivation is shown in the enclosed Supporting Information,
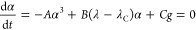
5where *g* is the magnitude of the gravitational field, and the remaining terms on the right-hand side of the equation can be understood as follows: *A*α^3^ is a nonlinear term that stabilizes the bifurcating states, *B*(λ – λ_C_)α may be regarded as a force that maintains the system’s symmetry, and *Cg* represents the difference in the rates at which the vertical and random branches of the chemical gardens form, with the coefficients *A*, *B*, and *C* determined from the chemical kinetic mechanism.

**Figure 3:**
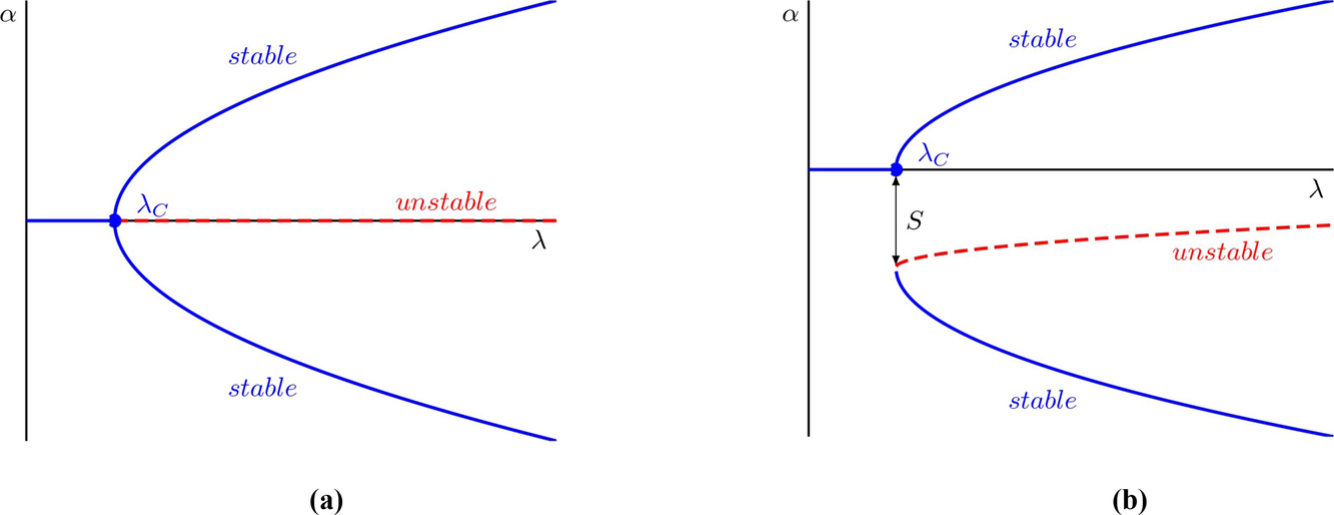
Sketch of a steady-state bifurcation diagram illustrating (a) symmetry in the absence of a gravitational field and (b) asymmetry in the presence of a gravitational field. The preferential evolution to the upper branch in (b) demonstrates gravity’s role in symmetry breaking.

Note that if *g* ∼ 0, eq 5 reduces to
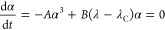
6whose corresponding bifurcation diagram is shown in Figure 3a. For λ < λ_C_, there is only one possible solution characterized by a very small amplitude α. However, at the critical point, α increases steeply, and two bifurcating solutions  are equally stable, while one remains unstable (α = 0). In turn, if *g* ≠ 0, eq 5 gives three different bifurcating solutions at the critical point, each with different probabilities of occurrence (Figure 3b). Likewise the previous case, for λ < λ_C_, only one stable solution exists, but as the constraint λ varies and reaches the critical point, three bifurcating solutions emerge: two stable solutions and one unstable solution. As λ becomes larger than λ_C_, the system preferentially evolves into the upper branch since a gravity-induced bias amplifies the probability of the upper branch solution over the lower one. In this case, a switch to the lower branch of the bifurcation diagram would only be possible if and only if the gravitational field is strong enough to overcome the fluctuations.

From Figure 3, it is clear that the quantity *S* depicted in Figure 3b depends on the magnitude of the gravitational field and the parameters *A*, *B*, and *C*. This quantity, referred to as nonequilibrium sensitivity, has an important physical meaning: it represents a measure of how effectively external fields can overcome fluctuations to produce observable and stable asymmetry between the bifurcating states as the system crosses the bifurcation point.

To estimate *S*, one should recall that eq 5 is a depressed cubic equation with all real coefficients and, consequently, it must have three real roots, with two of them being equal. Then, by calculating the difference between the two different real solutions of eq 5, one obtains the minimum separation *S* between the two bifurcation branches,^20^
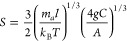
7where the term  is introduced to make *S* dimensionless, *m*_*a*_ is the atomic mass,  is the characteristic length of the system, *k*_B_ is the Boltzmann constant, and *T* is the thermodynamic temperature.

## 4 Results and Discussion

### 4.1 Nonequilibrium Sensitivity of Chemical Gardens to the Gravitational Field

The experimental evidence reported in Section 2 indicates that the differences observed in the formation and growth of chemical gardens under microgravity and normal gravity conditions are consistent with the behavior of a nonequilibrium chemical system undergoing a gravity-triggered bifurcation. In such systems, the interaction between concentration fluctuations and gravity results in a directional mass transport that destabilizes the solution **C**_0_ at the bifurcation point, leading to the formation of a specific macroscopic pattern.^26,27^ In other terms, when chemical gardens grow under 1**g** conditions, concentration fluctuations are amplified and become larger than the thermal fluctuations of the system. As a result, the system responds by favoring the formation of vertical metal silicate tubes over a more random pattern. Conversely, when chemical gardens grow under μ**g** conditions, the thermal fluctuations are larger than the concentration fluctuations, and the interaction between concentration fluctuations and gravity does not produce observable preferred direction effects.

Although the chemical kinetics and mechanisms of the precipitation reactions that lead to the formation of chemical gardens are still not well-known, it is possible to estimate the minimum strength of the gravitational field required to produce vertical chemical gardens. To this end, one recalls the important interplay between gravity and fluctuations near the bifurcation point, where the system undergoes a qualitative change in behavior. In fact, near the critical point, (i) the fluctuations are not negligible and the branch to which the system will evolve depends on the random fluctuations in the system and (ii) **Ψ** varies faster than α. In view of this, if one uses the adiabatic elimination principle,^28^ the dynamics of the slow variable α near the critical point are governed by a Langevin equation of the form,
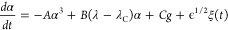
8where ϵ^1/2^ represents the random fluctuations in α, ξ(*t*) is a Gaussian white noise with zero mean (<ξ(*t*)> = 0) and no correlation (<ξ(*t*)ξ(*t′*)> = *Q*δ(*t* – *t′*)) that is regarded as an idealization of a real physical noise process, and *Q* is a parameter that depends on the chemical kinetics.^29^ Thus, it follows from eq 8 that the time evolution of the probability distribution *P*(α, *t*) is given by the following Fokker–Planck equation,
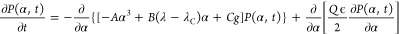
9whose steady-state solution is
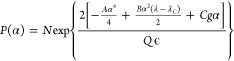
10where *N* is a normalization constant.

Equations 8, 9 and 10 are valid in the vicinity of the critical point λ_C_, where the deviations from the steady state are small and *P*(α, *t*) is essentially Gaussian (in fact, near the bifurcation point *P*(α, *t*) begins to deviate from its Gaussian form; nevertheless, because the relaxation time required for the probability distribution to reach its non-Gaussian steady state is very long, *P*(α, *t*) can be approximated as Gaussian as a first-order approximation^30,31^). In this region, α exhibits random fluctuations within the range of accessible values, spending most of its time at the values corresponding to the maxima of *P*(α), that is, the stable branches of the bifurcation diagram depicted in Figure 3b. Then, if the gravitational field is strong enough to overcome the fluctuations, as λ approaches λ_C_, the system will tend to preferentially evolve to one of the branches, although fluctuations can momentarily disrupt this pattern.

The arguments above may be now used to estimate the minimum strength of the gravitational field required to produce vertical chemical gardens. Let α_+_ and α_–_ be the values at which *P*(α) has its maxima. Moreover, let the ratio *P*(α_+_)/*P*(α_–_) be regarded as the ratio of time that α spends in the neighborhood of α_+_ and α_–_. If this ratio is evaluated when the separation between the branches α_+_ and α_–_ is minimum, that is, when two of the roots of eq 5 are coincident, one has
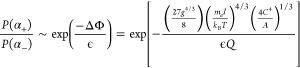
11where ΔΦ is a Boltzmann-like potential related to the probability difference between the two stable branches. Then, if ΔΦ/ϵ > 10 as usually assumed for a good bifurcation branch selection,^20,21^ it follows that
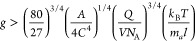
12where the fluctuations ϵ are on the order of (*V N*_A_)^−1^, *V* is the volume, and *N*_A_ is the Avogadro number.

By considering the typical experiment conditions, chemical gardens with structures on the order of a few centimeters  can be obtained in small vessels of *V* ∼ 2.0 × 10^–5^ m^3^ at room temperature (∼300 K). In addition, based on the values of kinetic rates observed for moderately fast precipitation reactions, the values of *A* and *C* are assumed to be on the order of ∼10^–4^, whereas *Q* is assumed to be ∼10^–3^ m^3^ mol^–1^, since *Q* is on the order of the fastest step of the kinetic mechanism. Therefore, with *m*_*a*_ = 1.6605 × 10^–27^ kg, *k*_B_ = 1.3806 × 10^–23^ J K^–1^, and *N*_A_ = 6.0221 × 10^23^ mol^–1^, one obtains
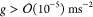
13

The above result shows that chemical gardens are sensitive to gravitational fields with a magnitude greater than . At this threshold, *S* becomes large enough to preferentially select one bifurcation branch over the other, leading to the formation of mostly vertical chemical gardens. Note, however, that under the conditions considered, eq 13 holds even if multiplicative noise—fluctuations in *A* and λ—is included in eq 8. In fact, since α is small in the vicinity of the critical point, time evolution of α is mainly determined by the last two terms on the right-hand side of eq 8, namely, *Cg* and ϵ^1/2^ξ(*t*).

## 5 Concluding Remarks and Future Perspectives

Motivated by the experimental results showing the differences in the formation and growth of chemical gardens produced via the seed growth method under microgravity (μ**g**) and normal gravity (1**g**) conditions, a nonequilibrium sensitivity analysis is performed in this work. To this end, chemical gardens are regarded as chemical systems far from equilibrium, whose dynamics are described by a solution-diffusion model with reaction. Beyond estimating the minimum strength of the gravitational field necessary to favor the formation of upward-growing chemical gardens, the results of the nonequilibrium sensitivity analysis suggest that the growth of chemical gardens is an experimental demonstration of symmetry breaking in chemical systems far from equilibrium.

**Figure 4:**
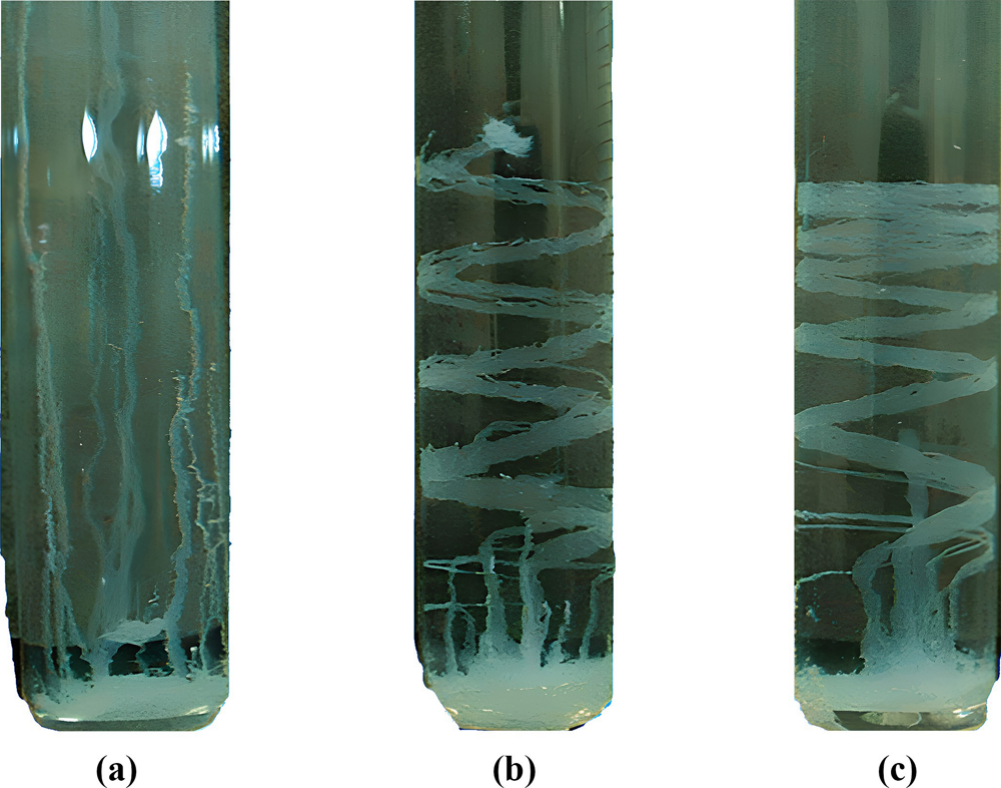
Zinc silicate chemical gardens produced via the seed method in the (a) absence of a magnetic field and presence of a magnetic field (b) 6 T and (c) 13.5 T.^33^ Adapted with permission from J. Phys. Chem. B 2004, 108, 8, 2527–2530. © 2005 American Chemical Society.

Although this paper focuses on the influence of the gravitational field on the formation and growth of chemical gardens, the theoretical framework presented here can also be applied to understanding how magnetic fields induce asymmetrical chiral growth in chemical gardens.^1^ When chemical gardens are produced in the absence of a magnetic field using the seed method, the precipitate grows upward as usual. However, in the presence of a downward magnetic field, the metal silicate tubes exhibit upward helical growth with right-handed chirality (Figure 4). Conversely, when the direction of the magnetic field is reversed, the metal silicate tubes still grow helically upward but with left-handed chirality.^32−34^ These findings provide additional experimental evidence of spontaneous symmetry breaking in chemical gardens, which merits further investigation in the context of nonequilibrium thermodynamic sensitivity theory.

Regarding the experiments reported in Section 2, gravity is not essential for the formation and growth of chemical gardens, but it is necessary to induce their upward growth. Indeed, the rupture of the metal silicate membrane plays a fundamental role in the mechanism of formation and growth of chemical gardens since this step is marked by the release of the pressure inside the membrane and a gravity-driven flow of metal ions toward the sodium silicate solution. At this moment, concentration fluctuations are enhanced by buoyancy forces and might become larger than the thermal fluctuations of the system.

Hence, the onset of the membrane rupture characterizes the moment at which λ > λ_C_, destabilizing the steady-state solution of the system. If the gravity-induced concentration fluctuations overcome the thermal fluctuations near the bifurcation point—a situation that should occur if the magnitude of the gravitational field is greater than  for the typical experimental conditions used in the synthesis of chemical gardens via the seed method—chemical gardens tend to grow as vertical tubes; otherwise they grow randomly.

## Supporting Information Available

Derivation of the reduced bifurcation equation with *g* ≠ 0 (eq 5) (PDF)
