# Supplementary material for: Gravity-Induced Symmetry Breaking in Chemical Gardens
Source: ACS Omega. 2025 Jan 28;10(9):9496–502. doi: 10.1021/acsomega.4c10551 (PMC11904849; doi:10.1021/acsomega.4c10551)
Supplement: Supplementary file 1 — ao4c10551_si_001.pdf [file ao4c10551_si_001.pdf]

Gravity-induced symmetry breaking in chemical gardens

Martina Costa Reis*

School of Engineering, University of São Paulo, 05508-010, São Paulo, Brazil

E-mail: martinacreis@usp.br

As stated in the manuscript, at the bifurcation point, the steady-state solution of the non-linear operator $\mathbf{N}(\mathbf{C}, \mathbf{g}, \lambda)$ is $\tilde{\mathbf{C}} = \mathbf{C_0} + \alpha\mathbf{\Psi}$. By substituting $\tilde{\mathbf{C}} = \mathbf{C_0} + \alpha\mathbf{\Psi}$ into

$$\frac{\partial \mathbf{C}}{\partial t} - \mathbf{D}\nabla^2\mathbf{C} + \boldsymbol{\eta}\mathbf{g}\cdot\nabla\mathbf{C} - \mathbf{F}(\mathbf{C}, \lambda) = \mathbf{0}\,, \tag{S.1}$$

one has,

$$\frac{\partial(\mathbf{C_0} + \alpha\mathbf{\Psi})}{\partial t} - \mathbf{D}\nabla^2(\mathbf{C_0} + \alpha\mathbf{\Psi}) + \boldsymbol{\eta}\mathbf{g}\cdot\nabla(\mathbf{C_0} + \alpha\mathbf{\Psi}) + \mathbf{F}(\mathbf{C_0} + \alpha\mathbf{\Psi}, \lambda) = \mathbf{0}\,. \tag{S.2}$$

This equation can be further simplified, if one considers that

$$\mathbf{N}(\mathbf{C_0}, \mathbf{g}, \lambda_C) = -\mathbf{D}\nabla^2(\mathbf{C_0}) + \boldsymbol{\eta}\mathbf{g}\cdot\nabla(\mathbf{C_0}) + \mathbf{F}(\mathbf{C_0}, \lambda_C) = \mathbf{0}\,, \tag{S.3}$$

and the mass production term is expanded around $\mathbf{C_0}$ and λ_C:

$$\mathbf{F}(\mathbf{C_0}, \lambda) = \mathbf{F}(\mathbf{C_0}, \lambda_C) + \left.\frac{\partial \mathbf{F}}{\partial \mathbf{C}}\right|_{\mathbf{C_0},\lambda_\mathbf{C}} \alpha\mathbf{\Psi} + \left.\frac{\partial \mathbf{F}}{\partial \lambda}\right|_{\mathbf{C_0},\lambda_\mathbf{C}} (\lambda - \lambda_C)\,\mathbf{\Psi} + \left.\frac{\partial^2 \mathbf{F}}{\partial \mathbf{C}^2}\right|_{\mathbf{C_0},\lambda_\mathbf{C}} \alpha^2\mathbf{\Psi}^2 + \ldots\,. \tag{S.4}$$

In this case, it follows that

$$\boldsymbol{\Psi}\frac{d\alpha}{dt}+\alpha\underbrace{\left[-\mathbf{D}\nabla^{2}+\left.\frac{\partial\mathbf{F}}{\partial\mathbf{C}}\right|_{\mathbf{C_0}}\right]}_{\mathbf{N^C}}\boldsymbol{\Psi}+\alpha\boldsymbol{\eta}\mathbf{g}\cdot\nabla\boldsymbol{\Psi}+(\lambda-\lambda_C)\left.\frac{\partial\mathbf{F}}{\partial\lambda}\right|_{\mathbf{C_0}}\boldsymbol{\Psi}+\ldots=\mathbf{0}\,, \tag{S.5}$$

where $\mathbf{N^C}$ is an operator that satisfies $\mathbf{N^C}\boldsymbol{\Psi}=\lambda\boldsymbol{\Psi}$. Hence, one has

$$\boldsymbol{\Psi}\frac{d\alpha}{dt}+\alpha\lambda\boldsymbol{\Psi}+\alpha\boldsymbol{\eta}\mathbf{g}\cdot\nabla\boldsymbol{\Psi}+(\lambda-\lambda_C)\left.\frac{\partial\mathbf{F}}{\partial\lambda}\right|_{\mathbf{C_0}}\boldsymbol{\Psi}+\ldots,=\mathbf{0}\,. \tag{S.6}$$

Note that the operator $\mathbf{N^C}$ is the Fréchet derivative of $\mathbf{N}(\mathbf{C},\mathbf{g},\lambda)$ with respect to $\mathbf{C}$. Hereafter, a similar notation will be used to express the higher-order derivatives that are necessary for the derivation of the reduced bifurcation equation.

The final form of the reduced bifurcation equation can be obtained by projecting each term of Equation (S.6) onto the adjoint of $\boldsymbol{\Psi}$. Here, one should recall that some quadratic terms in Equation (S.6) will be eliminated because $\boldsymbol{\Psi}$ satisfies the orthogonality condition with itself in a system that is invariant under spatial inversion. Thus, one obtains

$$\frac{d\alpha}{dt}=-A\alpha^{3}+B(\lambda-\lambda_C)\alpha+Cg=0\,, \tag{S.7}$$

where the constants A, B, and C are given by

$$A=\frac{1}{3!}\left\langle\mathbf{N^{CCC}}\boldsymbol{\Psi}\boldsymbol{\Psi}\boldsymbol{\Psi},\boldsymbol{\Psi}^{*}\right\rangle+\frac{1}{2!}\left\langle\mathbf{N^{CC}}(\mathbf{h_2}\boldsymbol{\Psi})+\mathbf{N^{CC}}(\boldsymbol{\Psi}\mathbf{h_2}),\boldsymbol{\Psi}^{*}\right\rangle\,, \tag{S.8}$$

$$B=\frac{1}{2!}\left\langle\mathbf{N^{CC}}(\boldsymbol{\Psi}\mathbf{h_1})+\mathbf{N^{CC}}(\mathbf{h_1}\boldsymbol{\Psi}),\boldsymbol{\Psi}^{*}\right\rangle+\left\langle\mathbf{N^{C\lambda}}\boldsymbol{\Psi},\boldsymbol{\Psi}^{*}\right\rangle\,, \tag{S.9}$$

$$C=\left\langle\underbrace{\boldsymbol{\eta}\mathbf{g}\cdot\nabla\boldsymbol{\Psi}}_{\mathbf{N^g}},\boldsymbol{\Psi}^{*}\right\rangle\,, \tag{S.10}$$

and

$$\mathbf{h}^1 = \mathbf{N}^{\lambda}\mathbf{N}^{\mathbf{C}^{-1}} \quad \mathbf{h}^2 = -\frac{1}{2!}\mathbf{N}^{\mathbf{C}^{-1}}\mathbf{N}^{\mathbf{CC}}\left(\mathbf{\Psi\Psi}\right) . \tag{S.11}$$

The mathematical procedure outlined above is also described in details in the papers about the non-equilibrium sensitivity theory[1,2].

References

(1) Kondepudi, D.; Prigogine, I. Sensitivity of non-equilibrium systems. *Phys.* **1981**, *107*, 1–24.

(2) Kondepudi, D.; Nelson, G. W. Chiral-symmetry-breaking states and their sensitivity in nonequilibrium chemical systems. *Phys.* **1984**, *125*, 465–496.
